# Downregulation of ADAM17 in pediatric immune thrombocytopenia impairs proplatelet formation

**DOI:** 10.1186/s12887-022-03237-x

**Published:** 2022-03-30

**Authors:** Qi Wang, Jia Wei, Xi Jia, Xiao Feng, Zhenghua Ji, Xueqiang Ji, Xuejun Shao

**Affiliations:** Department of Clinical Laboratory, Children’s Hospital of Soochow University, Suzhou, 215025 Jiangsu Province China

**Keywords:** Pediatric ITP, Megakaryocytes, ADAM17, Proplatelet formation

## Abstract

**Background:**

Immune thrombocytopenia (ITP) is the most common etiology of acquired thrombocytopenia diseases in children. ITP is characterized by the immune-mediated decreased formation and excessive destruction of platelets. The pathogenesis and management of pediatric ITP are distinct from adult ITP. A disintegrin and metalloproteinase 17 (ADAM17) mediates the shedding of platelet receptor glycoprotein Ib α (GPIb α) in extracellular domain, functioning in the platelet activation and clearance. Our study aims to probe the roles and mechanisms of ADAM17 in pediatric ITP.

**Methods:**

The differently expressed ADAM17 in megakaryocytes was obtained from children with ITP through the next-generation RNA-Sequence. Hematoxylin-eosin and Giemsa staining were performed for cell morphology identification. Flow cytometry was applied to assess autoantibodies against platelets, subtypes of lymphocytes, the surface expression level of ADAM17 and polyploidization of megakaryocytes, as well as the full-length GP Ib α.

**Results:**

ADAM17 was significantly downregulated in megakaryocytes and platelets in children with ITP. Higher values of PDW and positive autoantibodies presence were observed in children with ITP. Loss of ADAM17 in mice led to defects in proplatelet formation and significantly elevated expression of phosphorylated myosin light chain (p-MLC) in megakaryocytes.

**Conclusions:**

Our study indicated that the downregulation of ADAM17 might be an innate cause of inefficient platelet production in pediatric ITP.

**Supplementary Information:**

The online version contains supplementary material available at 10.1186/s12887-022-03237-x.

## Introduction

Immune thrombocytopenia (ITP) is a common heterogeneous autoimmune disease, characterized by the defective formation and excessive clearance of platelets (the platelet counts < 100 × 10^9^/L). The incidence of ITP in children varies from 2.2 to 5.3 per 100,000 individuals every year [1]. Although the definite pathogenesis of ITP is still unclear, the main mechanism is assumed to be caused by autoreactive destruction and the poor production of platelets [2–4]. The clinical guidelines and standard practices of pediatric ITP are different from those of adult ITP. Childhood ITP is often benign, and pediatric patients possess higher self-healing ability than adults [1]. However, pediatric ITP is strongly associated with high bleeding risks, as well as other short-term and long-term sequelae [5, 6]. The difference of bleeding manifestations between pediatric and adult ITP still needs to be noted.

A disintegrin and metalloproteinases (ADAMs), as membrane-bound enzymes, are major mediators of ectodomain shedding, function as the shedding or cleavage of various cell surface molecules [7]. Pathological dysregulation of expression and activity ADAMs are involved in many pathophysiological diseases, including tumorigenesis, infertility, and chronic immunity [8]. There are 13 human ADAM members known or predicted to be catalytically active, of which ADAM10 and ADAM17 are the most extensively studied [9]. Gardiner et al. [10] demonstrated that surface cleavage of platelet glycoprotein (GP) VI and platelet receptor Glycoprotein Ib α (GP Ib α) may be controlled by distinct mechanisms involving ADAM10 and/or ADAM17. The ectodomain of GP Ib α is shed by ADAM17, which mediates the formation of a non-covalent complex with GP Ib β and GP IX for platelet adhesion to collagen [11–16]. In addition, ADAM17 is also thought to be related to in vitro megakaryocyte formation [17]. The deletion of Zn^2+^ binding domain in ADAM17 (ADAM17 ^Zn△/ Zn△^) mouse leads to embryonic death with abnormal development in eyes, limbs and bleeding in fetus [18].

Despite the significant role of ADAM17 in platelet activation, the mechanism of ADAM17 in regulating platelet formation remains unclear. In this study, we found that ADAM17 ^Zn△/ Zn△^ megakaryocytes exhibited normal development but poor proplatelet formation and higher expression of p-MLC. The results indicate that ADAM17 is important in thrombopoiesis, and low expression of ADAM17 is possibly related to the observed thrombocytopenia in pediatric ITP.

## Methods and materials

### Patients

A total of 37 ITP children (age: 3.60 ± 3.64 years old; Male: 27) and 19 healthy donors (age: 4.60 ± 4.59 years old; male: 14) in the Children’s Hospital of Soochow University were enrolled from March 2019 to July 2020. All included subjects met the published criteria for ITP [19]. Platelet counts were lower than 100 × 10^9^/L, and platelet-forming megakaryocyte counts in bone marrow decreased in all patients. Furthermore, no splenomegaly has been found in patients. Children with any other hematological disease were excluded. All human samples were collected before any therapeutic procedures, including surgery and chemotherapy. Clinical and laboratory data were recorded, including patient age, gender, platelet counts, mean platelet volume (MPV), and platelet distribution width (PDW). All parents/local guardians were given an informed consent and agreed to participate. The study was approved by the Medical Ethics Committee of the Children’s Hospital of Soochow University and done in accordance with the Helsinki Principles.

### Next-generation RNA-Seq

Megakaryocytes from ITP children or healthy donors were purified by a single-step gradient solution (1.5%/3% bovine serum albumin) for RNA-Seq. In brief, RNA was prepared for sequencing using Ribo-Zero™ rRNA removal Kit (Illumina, San Diego, CA) for poly-A RNA. Base pairs of four children with ITP and four healthy controls were sequenced on an Illumina sequencer. Then libraries with different indices were multiplexed and loaded on an Illumina HiSeq instrument according to manufacturer’s instructions (Illumina). Sequencing was carried out using a 2 × 150 bp paired-end (PE) configuration; image analysis and base calling were conducted by the HiSeq Control Software (HCS) + OLB + GAPipeline-1.6 (Illumina) on the HiSeq instrument. The sequences were processed and analyzed by GENEWIZ.

### Giemsa staining of bone marrow smears

Bone marrow smears were fixed with icy methanol for 30 s, and then covered with 1 × Giemsa working solution at room temperature for 15 min. Smears were rinsed with deionized water for 3 min and were inspected under an inverted microscope. Megakaryocytes were captured under 1000× magnification using an Olympus BX 40 microscope. For each slice, at least 5 random megakaryocytes were selected for examination.

### Fetal liver cell culture and megakaryocyte purification

ADAM17^Zn△/+^ chimeric mice were purchased from Amgen Biologicals (Thousand Oaks, CA). The embryos were isolated at day 14.5 of pregnancy, and each fetal liver was genotyped and syringed into a single-cell suspension through a 100 μm filter. Cells were cultured in Dulbecco’s modified Eagle medium supplemented with 10% FBS, 1% penicillin, 1% streptomycin and purified, recombinant mouse TPO (50 ng/ml) at 37 °C and 5% CO_2_ for 4 days. On the fourth day, mouse fetal liver cells were allowed to sediment in a single-step gradient solution (1.5%/3% bovine serum albumin) for 50 min to purify mature megakaryocytes.

### Fetal liver histology

Fetal livers of ADAM17 ^Zn△/ Zn△^and ADAM17^+/+^ embryos were fixed overnight in 4% paraformaldehyde/PBS. Sections of paraffin-embedded tissues were stained using H&E kit (Beyotime, cat No. C0105, Haimen, China) followed by analysis and quantification. In brief, a total of eight mice from each strain were used, and over five sections from a similar location of each fetal liver were obtained. Ten random views were selected for microscopic examination and quantification for each section.

### Assays of proplatelet formation

Purified megakaryocytes were seeded at a density of 5 × 10^5^ cells per well in 24-well plates. Quantification of proplatelet-forming megakaryocytes was determined by the number of megakaryocytes with more than two pseudopods per well examined under bright field microscopy.

### Flow cytometry

For ploidy analysis, 75% cold ethanol pre-fixed megakaryocytes were treated with 0.02 mg/ml RNase A (Beyotime, cat No.ST576, Haimen, China) for 30 min at 37 °C and then double-stained with 0.01 mg/ml propidium iodide (Sigma Aldrich, cat No.P4170, USA) and FITC-conjugated rat anti-mouse CD41 antibody (BD Biosciences, cat No.553848, USA) for 30 min at room temperature. CD41^+^ cells were selected to assess ploidy. To quantify ADAM17, platelets were labeled with FITC-conjugated mouse anti-human CD41 antibody (BD Biosciences, cat No.555466, USA), and PE-conjugated mouse anti-human ADAM17 antibody (R&D, cat No. FAB9301P, Minneapolis, USA) for 30 min. A FACS (BD FACS Canto II, BD Biosciences, California 95,131, USA) flow cytometer was used for the flow cytometric analysis. Platelets (CD41^+^ population in whole blood) were selected to assess the expression of ADAM17. For autoantibodies analysis, the lysed platelets were incubated with auto-fluorescent microbeads, the microbeads coated with antigen cocktails (FITC conjugated GPIb/IX, GPIIb/IIIa, GMP 140; Changzhou Fucheng, cat No.CZFL001, China) were identified by different intensity of APC, “positive” was beyond to the cut off value of fluorescence intensity. Calculation formula of each cut off value (c.o.): c.o.(GP Ib) = 1.9× (MFI of FITC in controls), c.o.(GP IX, GP IIb, GP IIIa, GMP 140) = 1.5× (MFI of FITC in controls), MFI represents the mean fluorescence intensity [20].

### Western blot

Megakaryocytes were lysed in 1 × RIPA lysis buffer (Beijing Solarbio Science & Technology, cat No.R0010, Beijing, China) supplemented with a fresh protease and phosphatase inhibitor cocktail. The protein concentration was determined using a protein assay kit (Beyotime Biotechnology, cat No.P0010, Haimen, China). SDS PAGE electrophoresis was used to separate total proteins and nitrocellulose membranes were applied for protein transfer. After being blocked with 5% non-fat milk, membranes were incubated with specific primary antibodies at 4 °C overnight, followed by 1 h of further incubation with goat anti-rabbit IRDye 800CW or goat anti-mouse IRDye 800CW secondary antibodies (LI-COR Biosciences, Lincoln, NE) at room temperature. The membranes were visualized with Infrared Imagine System (LI-COR Biosciences, Lincoln, NE). The primary antibodies used in this study were rabbit-anti-P-Myosin light chain (1:500, Cell Signaling, cat No.3671, USA), and mouse-anti-mouse β-actin (1:1000, Beyotime, cat No.AA128, Haimen, China). The densitometry measurements of scanned blots were assessed using Image J software (NIH, Maryland, USA).

### Statistical analysis

Data analysis was subjected to SPSS version 24.0 (IBM Corp, USA) and GraphPad Prism Software (San Diego, USA). Two-tailed Student’s t test was used for comparisons between 2 groups. One-way ANOVA followed by Bonferroni post-hoc test was used for multiple-group comparisons. Besides, Spearman’s correlation analysis was used to assess the relationship between MFI of ADAM17 and other laboratory test indicators. A *P* value less than 0.05 was considered statistically significant.

## Results

### Downregulated ADAM17 in children with ITP

**Fig. 1:**
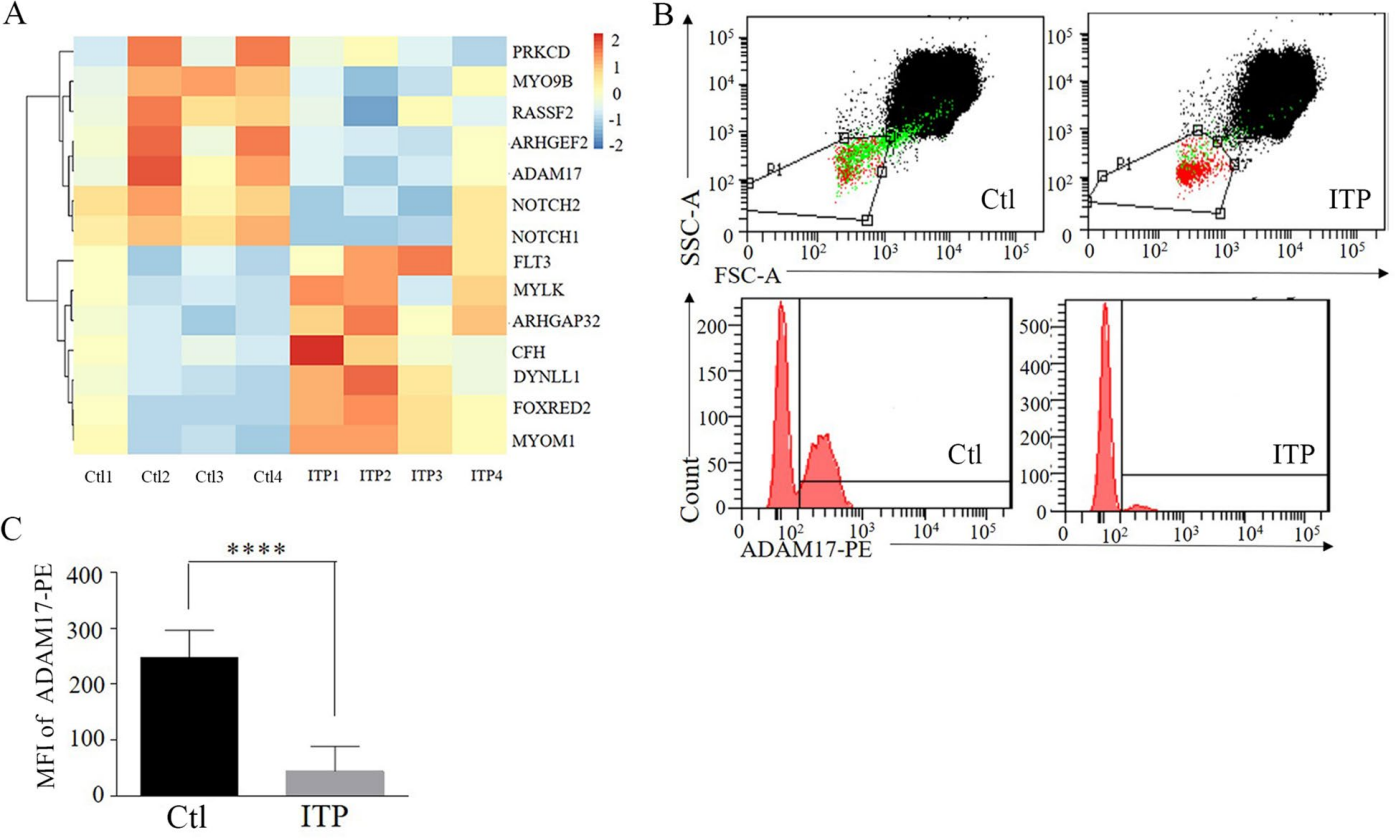
Expression of ADAM17 was downregulated in children with ITP. (**A**) Heat map of megakaryocytes following Next-generation RNA-Seq of whole exons. The study shows part results of altered gene pattern in children with ITP (*n* = 4) and healthy control (*n* = 4). (**B**) Flow cytometry analysis of ADAM17 expression on peripheral blood platelets. The gated domain contains platelets. (**C**) Quantification of the MFI of PE-conjugated ADAM17 antibody among children with ITP (*n* = 16) and healthy control (*n* = 17). Data were shown through Mean ± SD; Ctl indicated healthy control children. ITP indicated children with immune thrombocytopenia; ***P<0.001; ****P<0.001

We found decreased expression of *ADAM17*, Notch and cytoskeleton related genes (*ARHGEF* and *MYO9B*) among ITP children through the RNA-seq of bone marrow megakaryocytes. In contrast, the expression of *ARHGAP, MYOM1,* and *MYLK* were elevated (Fig. 1A). The expression of platelet *ADAM17* from children with ITP (ITP 44.5 ± 18.1) was also obviously declined compared to the control group (Ctl 248.1 ± 18.3) (*P<0.0001*) (Fig. 1B and C).

**Fig. 2:**
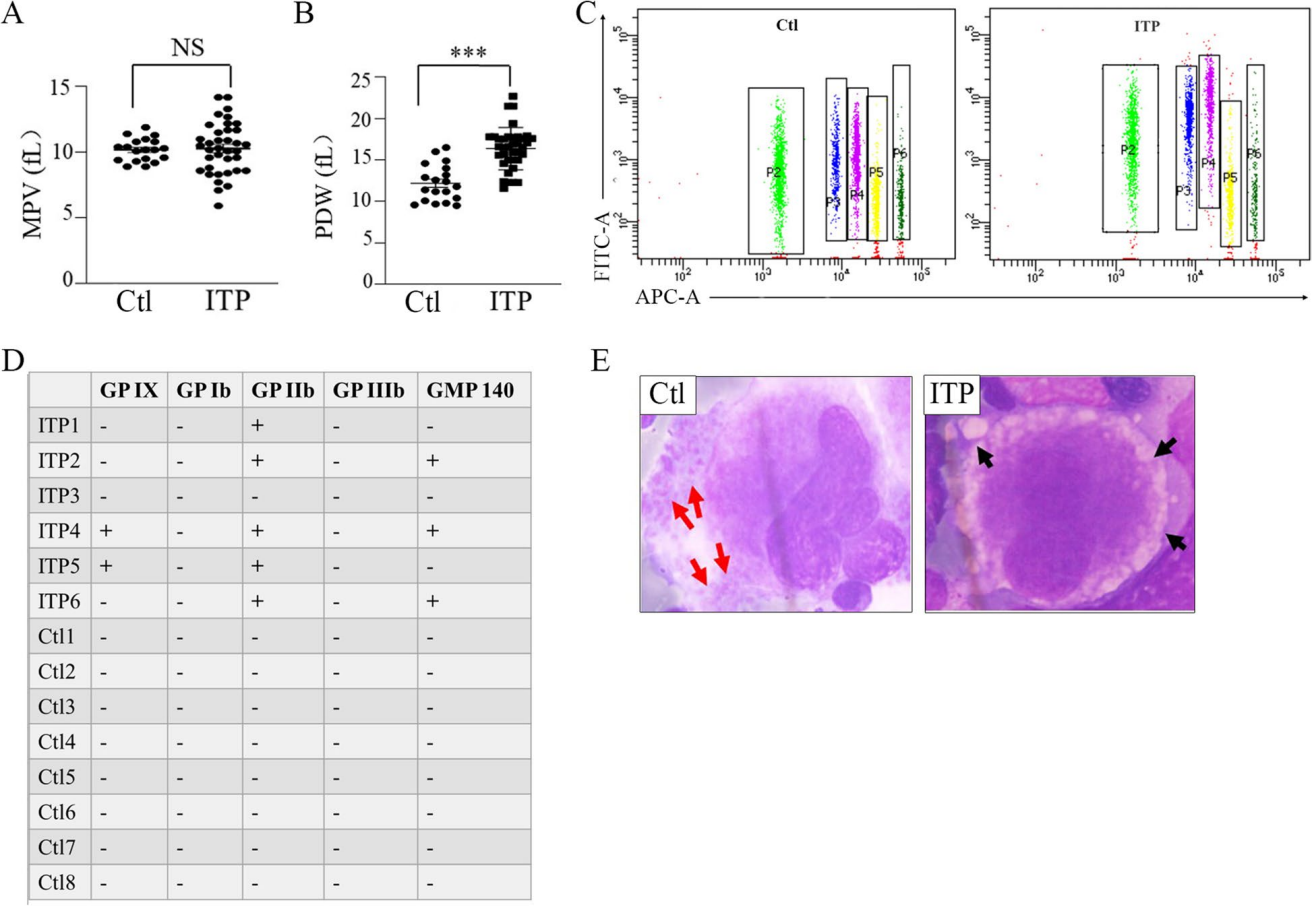
Detection of platelet-related indicators. (**A**) Quantification of PDW in children with ITP (*n* = 37) and healthy control (*n* = 19). (**B**) The expression level of MPV was measured in children with ITP (*n* = 37) and healthy control (*n* = 19). (**C**) Flow cytometry analysis of antibodies against platelet in children with ITP (*n* = 6) and healthy control (*n* = 8). P2, P3, P4, P5 and P6 represents GP IX, GP Ib, GP IIb, GP IIIa and GMP 140, respectively. (**D**) Positive calculated results of flow cytometry. (**E**) Giemsa staining of bone marrow smears. Red arrows indicated new-born platelets from healthy control megakaryocytes, and black arrows indicated vacuoles from ITP megakaryocytes. The magnification was 1000 folds each slice. Three random megakaryocytes were selected for examination. ITP indicated children with immune thrombocytopenia; Ctl indicated healthy control; NS indicated not significantly different

In addition, children with ITP had significantly higher PDW levels than healthy donors (Mean ± SD: Ctl 12.2 ± 2.2; ITP 16.4 ± 2.6) (*P*<0.001) (Fig. 2A), and there was no significant difference in MPV (Fig. 2B), suggesting the abnormal production of platelets in patients. Platelet autoantibodies are mainly directed against the platelet glycoproteins in ITP. Results of the cytometric bead array showed that positive reactions of GP IX, GMP 140, GP IIb in children with ITP were 20.0, 33.3, and 50.0%. No autoantibodies were detected in the control group (Fig. 2C and D). Furthermore, the bone marrow megakaryocytes from children with ITP showed impaired proplatelet formation and had more vacuoles rather than newborn platelets (Fig. 2E). The sizes or maturation of the nucleus were still normal. The MFI of ADAM17 was negatively associated with PDW (*R* = -0.552, *P =* 0.002) while positively associated with the count of CD4+ T cells (*R* = 0.562, *P* = 0.001) and the ratio of CD4+/CD8+ T cells (*R* = 0.629, *P* < 0.001).

### Normal cell size and ploidy of ADAM17 ^Zn△/ Zn△^ megakaryocytes

**Fig. 3:**
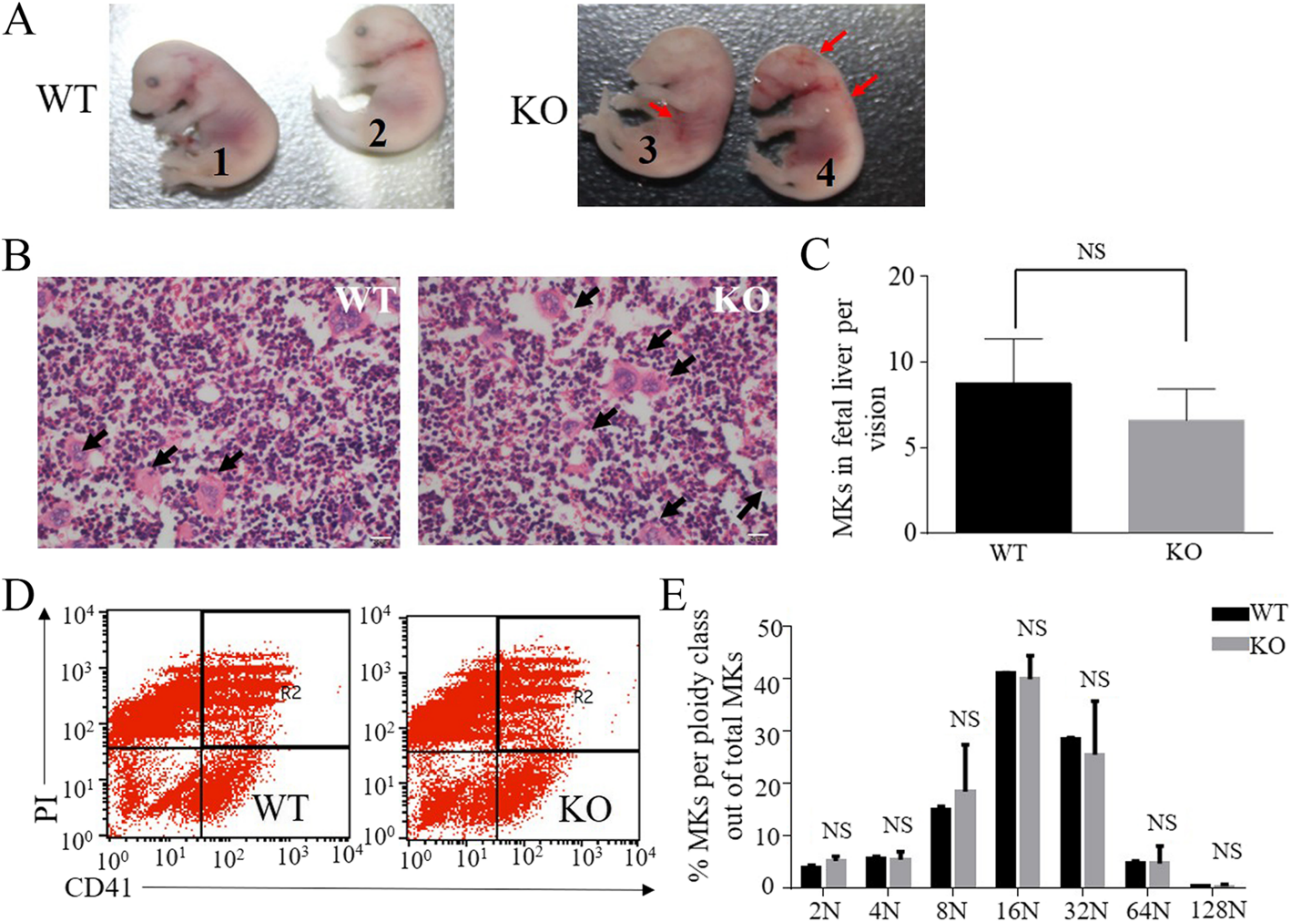
Normal cell size, ploidy of fetal liver-derived megakaryocytes from ADAM17 deficient mice. (**A**) The fetus of ADAM17^+/+^ (wildtype, WT, left) and ADAM17 ^Zn△/ Zn△^(knockout, KO, right) mice, red arrows indicate bleeding spot. (**B**) The H.E. staining of mouse fetal livers, black arrows indicate megakaryocytes. Scale bar, 40 μm. (**C**) Quantification of megakaryocytes in fetal livers (WT, *n* = 8; KO, *n* = 10). Mean ± SEM is shown. NS indicated not significantly different. (**D**) Flow cytometry analysis of megakaryocyte ploidy by CD41 and propidium iodide (PI) double staining. (**E**) The percentages of megakaryocytes per ploidy class out of total megakaryocytes were determined using flow cytometry analysis, cell counts were indicated by propidium iodide staining. Data are Mean ± SD (*n* = 3 per arm)

To determine the function of ADAM17 in platelet production, we used *ADAM17* knock out fetal livers at day 14.5 (E 14.5). ADAM17 ^Zn△/ Zn△^ mice had defective in eyes and limbs (Fig. 3A). The fetus of ADAM17 ^Zn△/ Zn△^ mice exhibited severe thrombocytopenia with bleeding spots in head, neck and abdomen (Fig. 3A). The H.E. staining of mouse fetal livers showed no difference in cell size (Fig. 3B) and cell numbers (WT 8.75 ± 0.92; KO 6.6 ± 0.58) (Fig. 3C). The induced megakaryocytes from ADAM17 ^Zn△/ Zn△^fetal liver suspension cells had normal ploidy distribution (2 N: WT 4.15 ± 0.18, KO 5.25 ± 0.37; 4 N: WT 5.91 ± 0.15, KO 5.56 ± 0.61; 8 N: WT 14.98 ± 0.28, KO 16.86 ± 4.0; 16 N: WT 41.1 ± 0.06, KO 40.12 ± 1.87; 32 N:WT 28.44 ± 0.14, KO 25.6 ± 4.18; 64 N:WT 4.89 ± 0.16, KO 4.71 ± 1.38;≥128 N: WT 0.49 ± 0.01, KO 0.36 ± 0.17) (Fig. 3D and E).

### Decreased proplatelet-formation by ADAM17 ^Zn△/ Zn△^ megakaryocytes

**Fig. 4:**
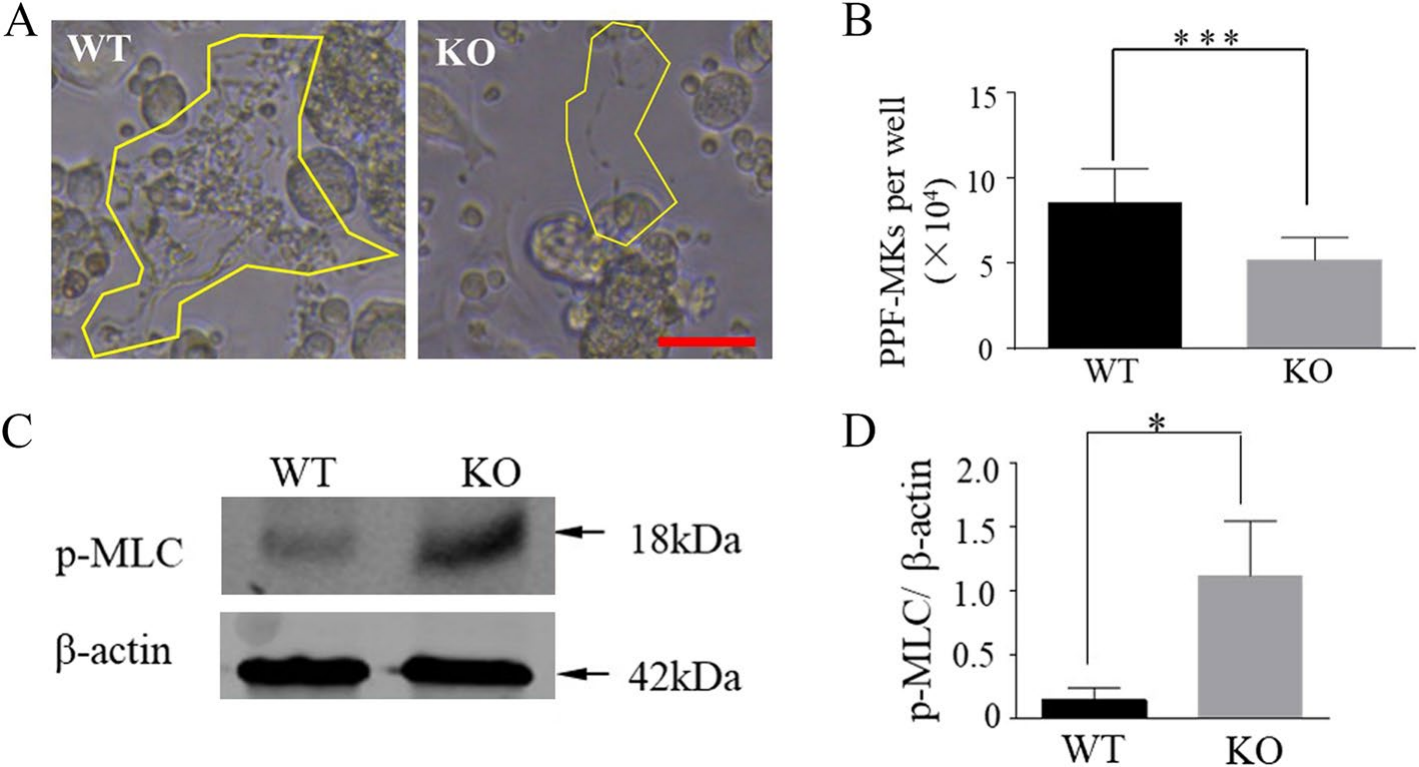
Decreased proplatelet formation by ADAM17^Zn△/Zn△^megakaryocytes. (**A**) Representative images of mouse fetal liver-derived megakaryocytes producing proplatelets. Yellow border indicates a megakaryocyte undergoing proplatelet formation. Scale bar, 25 μm. (**B**) Quantification of proplatelet-forming megakaryocytes. Mean ± SD (*n* = 5 per arm). (**C**) Immunoblots of p-MLC expression in megakaryocytes. (**D**) p-MLC expression normalized to β-actin by densitometry analysis. Data are Mean ± SD (*n* = 5 per am). *P<0.05, ***P<0.001

Though the size and polyploidization of ADAM17 ^Zn△/ Zn△^ megakaryocytes were normal, the number of protruding barbell-like proplatelets in ADAM17 ^Z△/ Zn△^ megakaryocytes were significantly decreased (Fig. 4A and B). The cytoskeleton is vital for the proplatelet elongation and bifurcation, and increased phosphorylation of myosin light chain (p-MLC) could suppress cytoskeleton organization [16]. We found the expression of p-MLC in ADAM17 ^Zn△/Zn△^ megakaryocytes was significantly higher than that in ADAM17^+/+^ megakaryocytes (Fig. 4C and D). The full-length gels and blots are included in a Supplementary Fig. S2.

## Discussion

The deficit in proplatelet formation leads to low platelet counts in ITP [21]. In this study, ADAM17 was downregulated in megakaryocytes and platelets in children with ITP. The loss of ADAM17 in mouse fetal livers did not affect the number and polyploidization of derived megakaryocytes but decreased the release of proplatelets.

The thrombocytopenia in ITP is caused mainly by the production of autoantibodies against the GP Ib-IX and GP IIb-IIIa complex, leading to platelet consumption through over-activation of macrophagocytes [22]. Autoantibodies can also lead to poor platelet production by interfering with megakaryopoiesis [23]. The high level of PDW in children with ITP indicates the low quality of platelet production. In the chronic inflammatory autoimmune disorder, Sjögren’s syndrome (SS), autoantibodies activate the TACE/TNF-a/NF-kB axis to exert their pathogenic effects [24]. Therefore, the relation between ADAM17 and autoantibodies in children with ITP should be taken into consideration in our further study. Additionally, the reduced regulatory T cells in ITP could decrease the immunosuppressive effect on B cells (plasma cells) and severe the accumulation of CD8^+^ cytotoxic T cells, resulting in direct platelet lysis [25]. Consistent with this, we observed the elevated expression of CD8^+^ T cells in ITP children, while the count of CD4^+^ T cells was decreased in our study.

ADAM17 is a wide-substrate sheddase involved in cleavage of numerous cytokines and membrane antigens [26]. This cleavage process can release soluble molecules that exert agonistic or antagonistic functions. ADAM17 is associated with various pathologies, including cardiovascular diseases, cancers, acute inflammatory diseases [27]. Previous studies showed that the plasma from adult patients with ITP contained increased levels of GPIb α fragments shed by upregulated ADAM17 of platelets [28, 29]. However, we found the decreased ADAM17 level of megakaryocytes and platelets and increased full-length GPIb α of platelets from children with ITP (Supplementary Fig. S1). The contradictory results may be because this study focuses on children with ITP rather than adults. Additionally, previous studies demonstrated that isolated platelets after being treated with extracted ITP plasma in vitro led to increased activity and expression of metalloprotease ADAM17, which was different from conditions in vivo. In our murine experiments, the decreased activity of ADAM17 in fetal liver hematopoietic cells resulted in normal maturation of fetal liver-derived megakaryocytes. However, poor in vitro proplatelet formation indicated that ADAM17 is important in thrombopoiesis. As another vital sheddase of GPIbα, ADAM10 displayed no change by RNAseq (data was not shown), supporting the more important role of ADAM17 in pediatric ITP. The cause of lower expression of ADAM17 in platelets would be related to its maturation from endoplasmic reticulum (proADAM17, the immature zymogen of ADAM17) to Golgi body (mADAM17, the mature form of ADAM17) [30].

Research about bortezomib-induced thrombocytopenia during relapsed multiple myeloma therapy showed the accumulation of RhoA in megakaryocytes enhanced phosphorylation of myosin light chain and inhibited proplatelet formation [31]. Cytoskeleton arrangement is vital in proplatelet formation, and Rho GTPase is a key switch. At the early stage of megakaryopoiesis, RhoA prevents actomyosin accumulation to facilitate megakaryocyte polyploidization, whereas it is downregulated gradually at the late stage of megakaryopoiesis [32–34]. We also found that increased expression of p-MLC in ADAM17 ^Zn△/Zn△^megakaryocytes supported aberrant cytoskeleton arrangement in megakaryocytes without ADAM17. TNF-α, one substrate of ADAM17, can regulate RhoA by different mechanisms in tubular epithelial cells [35]. The mechanism of ADAM17 in regulating megakaryocyte cytoskeleton arrangement and in thrombopoiesis may need to be explored in the further study of TNF-α and RhoA. Due to the limitations of follow up, the information on platelet ADAM17 expression from different childhood patients (the newly diagnosed, while in treatment or in remission) was not complete. Further studies on ADAM17 knocked in ADAM17 ^Zn△/Zn△^megakaryocytes and platelet-producing in vivo should be carried out.

In summary, the expression of ADAM17 is downregulated in pediatric ITP. Loss of ADAM17 in megakaryocytes does not affect megakaryopoiesis but impairs thrombopoiesis. Our findings provided a new pathway by which low platelet counts may occur in childhood ITP, which could be targeted to prevent the thrombocytopenia.

## Supplementary Information

**Additional file 1: Supplementary Figure S1.** Flow cytometry analysis of expression levels of ADAM17 and GP Ib α. (A) Results of flow cytometry analysis in healthy controls (*n*=8) and children with ITP(*n*=6). (B) Quantification of ADAM17 and GP Ib α by MFI.

**Additional file 2: Supplementary Figure S2.** Results of the full-length gels and blots of western blots referring to murine ADAM17 ^Zn△/ Zn△^megakaryocytes. (A) The full-length gels and blots of p-MLC. (B) The full-length gels and blots of β-actin.

## Data Availability

The datasets generated during and analyzed during the current study are not publicly available due to the further study would be based on these data but are available from the corresponding author on reasonable request.

## Abbreviations

ITP: Immune thrombocytopenia
ADAMS: A disintegrin and metalloproteinases
GPIbα: Platelet receptor glycoprotein Ibα
p-MLC: Phosphorylated myosin light chain
