# Supplementary figures and images for: Downregulation of ADAM17 in pediatric immune thrombocytopenia impairs proplatelet formation
Source: BMC Pediatr. 2022 Mar 30;22:164. doi: 10.1186/s12887-022-03237-x (PMC8966352; doi:10.1186/s12887-022-03237-x)

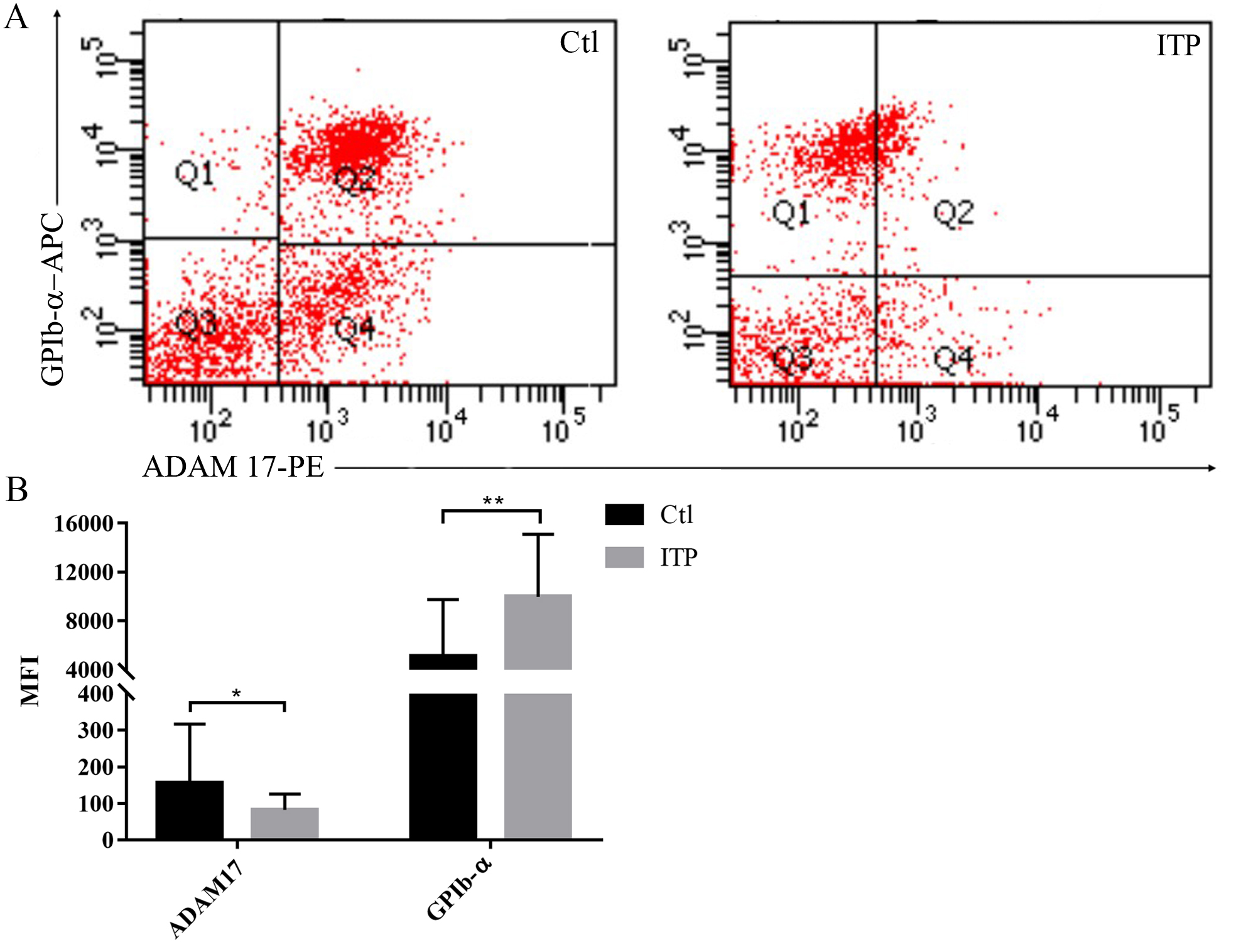

Supplement: Supplementary file 1 — Additional file 1: Supplementary Figure S1. Flow cytometry analysis of expression levels of ADAM17 and GP Ib α. (A) Results of flow cytometry analysis in healthy controls (n=8) and children with ITP(n=6). (B) Quantification of ADAM17 and GP Ib α by MFI. [file 12887_2022_3237_MOESM1_ESM.jpg]

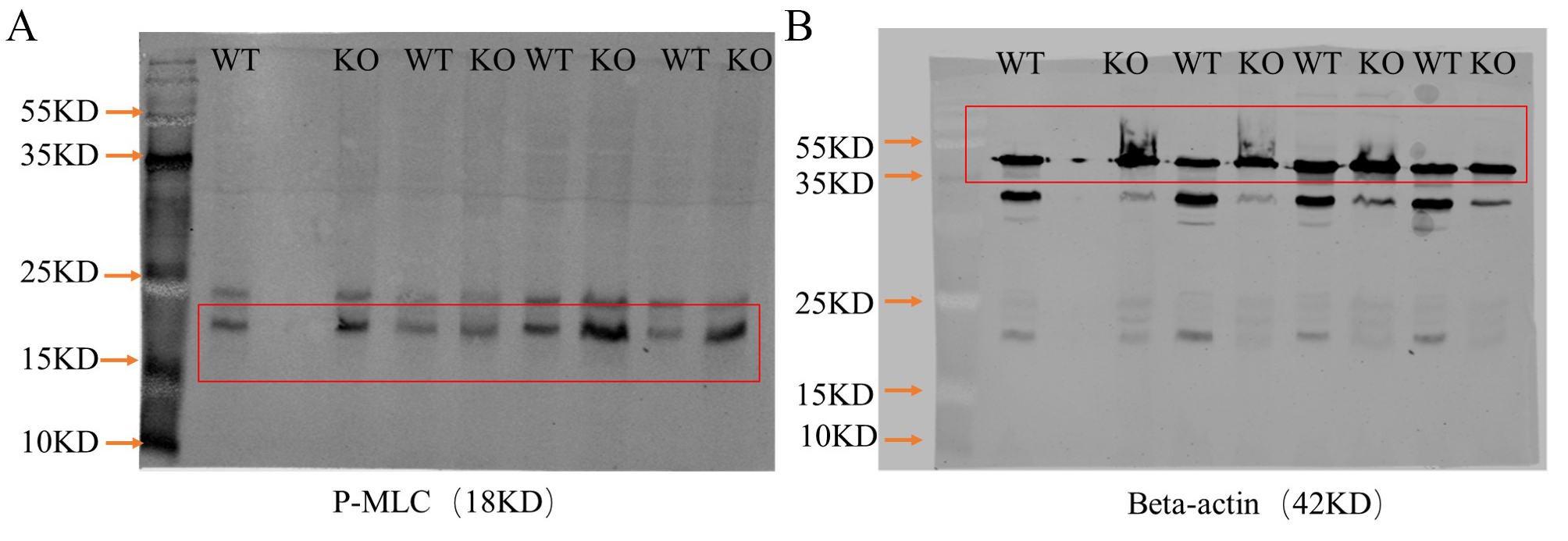

Supplement: Supplementary file 2 — Additional file 2: Supplementary Figure S2. Results of the full-length gels and blots of western blots referring to murine ADAM17 Zn△/ Zn△megakaryocytes. (A) The full-length gels and blots of p-MLC. (B) The full-length gels and blots of β-actin. [file 12887_2022_3237_MOESM2_ESM.jpg]
